# Motor Learning Deficits in Parkinson's Disease (PD) and Their Effect on Training Response in Gait and Balance: A Narrative Review

**DOI:** 10.3389/fneur.2019.00062

**Published:** 2019-02-07

**Authors:** Markey Olson, Thurmon E. Lockhart, Abraham Lieberman

**Affiliations:** ^1^Locomotion Research Laboratory, School of Biological and Health Systems Engineering, Arizona State University, Tempe, AZ, United States; ^2^Muhammad Ali Movement Disorders Clinic, St. Joseph's Hospital and Medical Center, Barrow Neurological Institute, Phoenix, AZ, United States

**Keywords:** Parkinson's disease, motor learning, gait and balance, motor training, postural learning, physical therapy, repeated perturbation training

## Abstract

Parkinson's disease (PD) is a neurological disorder traditionally associated with degeneration of the dopaminergic neurons within the substantia nigra, which results in bradykinesia, rigidity, tremor, and postural instability and gait disability (PIGD). The disorder has also been implicated in degradation of motor learning. While individuals with PD are able to learn, certain aspects of learning, especially automatic responses to feedback, are faulty, resulting in a reliance on feedforward systems of movement learning and control. Because of this, patients with PD may require more training to achieve and retain motor learning and may require additional sensory information or motor guidance in order to facilitate this learning. Furthermore, they may be unable to maintain these gains in environments and situations in which conscious effort is divided (such as dual-tasking). These shortcomings in motor learning could play a large part in degenerative gait and balance symptoms often seen in the disease, as patients are unable to adapt to gradual sensory and motor degradation. Research has shown that physical and exercise therapy can help patients with PD to adapt new feedforward strategies to partially counteract these symptoms. In particular, balance, treadmill, resistance, and repeated perturbation training therapies have been shown to improve motor patterns in PD. However, much research is still needed to determine which of these therapies best alleviates which symptoms of PIGD, the needed dose and intensity of these therapies, and long-term retention effects. The benefits of such technologies as augmented feedback, motorized perturbations, virtual reality, and weight-bearing assistance are also of interest. This narrative review will evaluate the effect of PD on motor learning and the effect of motor learning deficits on response to physical therapy and training programs, focusing specifically on features related to PIGD. Potential methods to strengthen therapeutic effects will be discussed.

## Introduction

Parkinson's disease (PD), a neurological disorder characterized by bradykinesia, rigidity, tremor and postural instability and gait disability (PIGD), is known mainly as a degeneration of the dopaminergic neurons of the substantia nigra pars compacta. Resulting inhibition of the direct pathway and excitation of the indirect pathway in turn excites the globus pallidus interna (GPi), which leads to inhibition of the thalamus and the pedunculopontine nucleus (PPN) (1). Up to 80% of dopaminergic neurons may be destroyed before a patient exhibits significant signs of PD. The loss of such a substantial portion of dopaminergic cells affects the cardinal motor symptoms of PD and contributes to non-motor symptoms, including autonomic dysfunction, cognitive and neurobehavioral abnormalities, and sleep disorders (1–3). While levodopa is extraordinarily helpful for some of the symptoms of PD, others, especially related to PIGD and non-motor features, are relatively unaffected by current pharmacological or surgical treatments (4–10), making physical therapy and motor training one of the most promising current options for improving these symptoms (11–16). However, research indicates that PD leads to degradation of motor learning, which could limit the benefits of therapy (17–24). To maximize the benefit that may be achieved, the mechanisms of learning affected by PD need to be understood and the strategies that best circumvent these difficulties be researched.

While the dopaminergic system is the one most frequently implicated in PD, other neurological systems are also affected, including the cholinergic, noradrenergic, glutaminergic, and GABAergic pathways (25–28). The cholinergic system in particular has been intensely researched recently, though motor learning literature specifically related to the PPN and other cholinergic centers is still sparse. Research supports the effect of cholinergic denervation on attention, fall history, levodopa resistance, more advanced symptoms, and non-tremor dominant subtypes of PD (24, 29–32). All of these factors have also been related to motor learning degradation, suggesting that the cholinergic system and possibly other affected systems may play a role in modulating learning behavior (33–40). Multifactorial influence helps to explain somewhat heterogenous results of studies of both baseline gait and balance and motor learning in PD (17–24). Understanding these interconnected pathways and their effects in PD can help to identify the aspects of gait, balance and motor learning that patients struggle with and help to proscribe therapies and treatments most likely to benefit.

This paper will discuss previous research into the effects of PD on motor learning, focusing on the types of learning affected, the severity of these deficits, and modifications that might negate some of these effects. Taking these factors into account, recent research regarding the ability of patients with PD to respond to physical, exercise, and repeated perturbation training (RPT) therapies will be reported, with specific focus given to those studies related to the training of gait and balance. Methods utilized for literature review and article selection are further detailed in the Appendix. Future directions for research into the mechanisms related to gait, balance, and motor learning in PD and potential training strategies to improve motor learning and retention will also be discussed.

## Motor Learning in PD

Studies have closely linked the striatal system to motor learning (22, 41, 42), suggesting that patients with PD would, in addition to the degradation of their movement patterns at baseline, have difficulty acquiring movement schema to learn tasks. However, studies examining the ability of patients with PD to learn and adapt to motor tasks have been somewhat inconsistent (11–24, 43–51). While studies indicate that individuals with PD are still able to learn motor tasks, there is disagreement about the amount and type of possible improvement. One explanation for this is that conflicting studies utilized different types of learning. Specific aspects of learning are more severely impacted by PD than others, especially in the early stages of the disease. It has been indicated that people with PD are able to learn specific tasks, though they may need more practice than healthy controls to do so, but that these skills are not easily generalizable to other tasks, even if those tasks are similar (22, 43–48). The slower rate of learning, lack of generalizability, and the difficulty individuals with PD exhibit in dual-tasking imply that people with PD are still able to learn in a feedforward manner, but that they are unable to easily adjust to changes requiring use of automatic or reactive mechanisms. Because of this inability, they have difficulty adapting to changing conditions or monitoring multiple tasks, both of which are often required for balance and gait. To better understand why this is this case, it is important to differentiate types of motor learning and how they are specifically affected by PD.

### Explicit vs. Implicit Learning

Existing literature about the deficits of persons with PD in explicit and implicit learning has been somewhat conflicting, due in part to differing experimental definitions of the terms. Implicit, or procedural learning, is generally defined as motor skill learning acquired incidentally through practice and is often considered to be due to unconscious or reactive mechanisms. Explicit learning, on the other hand (or declarative learning), is more intentional learning often defined as consciously learned. It is based on past similar experience and has previously referred to cognitive tasks, such as remembering lists of words, while motor tasks would have been grouped with procedural learning. Because of this, motor learning researchers, in adapting implicit vs. explicit learning definitions to describe aspects of motor learning, have often differed in exact usage, which has led to confusion about what aspects are affected in PD. In order to better clarify the types of learning affected by PD, this paper will discuss the results of papers using differing definitions of explicit vs. implicit learning separately and then attempt to unite these findings in a cohesive, multifaceted whole.

The most commonly adopted definition of implicit learning is the ability of subjects to learn a repeating task without awareness of the pattern (in comparison with ability to learn a randomized task), while explicit learning may be measured as the ability to improve in the context of a known pattern. Studies have indicated that implicit and explicit learning of this type utilize different neural pathways. Implicit learning is associated with basal ganglia structures while explicit learning is more closely associated with the medial temporal lobe, implying that patients with PD should have difficulty mainly with implicit learning (52–59). Most studies of motor learning in PD have found that this type of implicit learning is affected by PD, though whether this problem is an early symptom of the disease or a progressive complication is still unclear (20, 23, 24, 60–64). A recent study suggests that implicit learning involving the integration of multiple components is more affected by PD than single-component tasks (65), which might be related to attentional resources. There is some disagreement about whether explicit learning is spared in PD, with many studies finding that both implicit and explicit learning are affected to some extent (20, 61, 66). It is currently unknown to what degree potential explicit learning degradation is affected by factors, such as disease duration, degeneration of the cholinergic and other neurotransmitter systems, and comorbidities, such as dementia and cognitive decline. Studies appear to implicate PD in attenuation of both implicit and explicit learning processes defined in this matter. However, patients with PD still appear to be better able to operate in explicit than implicit conditions. While healthy controls completed a motor targeting task most accurately with minimal outside feedback, PD subjects were better able to complete the same task in the condition with the highest amount of feedback (67).

Other studies define implicit vs. explicit learning based on the amount of error present during learning trials. The generation of a large, sudden error is considered explicit learning while slow, gradual introduction of error, or restriction of error using guidance systems, is considered to induce implicit learning. This definition of learning found that patients were able to adapt to implicit changes but struggled with or were completely unable to adapt to explicit perturbations, especially in the context of other tasks. A visuomotor perturbation study utilizing reaching tasks found that individuals with PD adapted similarly to healthy controls during introduction of small, gradual visuomotor perturbations leading to minimal errors, but were slower to adapt and displayed lowered magnitudes of adaptation during larger perturbations (68). A study examining the ability of subjects to learn a hammering task either with or without a guidance system found that those that were constrained by the guidance system showed increased learning and learning automaticity, as evidenced by ability to translate the hammering motion to dual-taking conditions. However, it is uncertain whether the learning effects obtained through this method translate to non-guided motions upon the removal of the guidance system (69). This implies that slow, gradual initiation of perturbations may be more helpful for patients with PD, such as through the use of assisted or constrained training. However, these results may not be easily translated to conditions outside of training unless training conditions are slowly scaled to mirror them.

Together, these results indicate that people with PD, while able to learn, have difficulty creating and switching between motor sets. They may require explicit information and additional practice relative to healthy age-matched controls to do so. The use of augmented cues (19, 67) and gradual introduction of perturbation (68, 69) may help patients to create feedforward motor sets. Such cues may be especially helpful during conditions that require attention to be divided or require reaction to unexpected perturbations. The ability of individuals with PD to retain training effects is also contested (19), possibly due to inability to automate sets.

In addition to the above definitions of implicit vs. explicit learning, a related concept of feedforward vs. reactive control of movement patterns is often discussed. A distinction does need to be made. One need not be consciously aware of a feedforward adaptation, though feedforward movements may be less likely to be fully automatized, and reactive mechanisms, while more akin to implicit learning, can also be trained explicitly. For this reason, feedforward and reactive mechanisms will be discussed separately in the next section.

### Feedforward vs. Reactive Mechanisms

Patients with PD are thought to have major difficulties in staging reactive responses to motor perturbations. Bradykinesia, akinesia, reduced proprioceptive inputs, and other factors likely play a role in this disability. Impaired feedback mechanisms, commonly noted in PD, also play a role. Individuals with PD are known to have proprioceptive and tactile sensory deficits (70), leading to decreased feedback and increased noise and ambiguity during motor tasks. These deficits are thought to cause increased reliance on visual feedback and attention-based control (71). People with PD also have difficulty updating task information based on discrete feedback in decision-making trials (72), suggesting an inability to properly use feedback that is received. Patients with PD appear to be unable to correctly determine the importance of feedback and events (73), which likely contributes not only to deficits in learning but also to problems with attention and motivation.

However, it appears that an independent difficulty in creating, automatizing, and switching between motor sets is also involved. This progressive loss of automatic motor control increases reliance on goal-directed control mechanisms, even for habitual actions (22, 74). Many studies have found defects in reactive responses and responses to novel scenarios in PD but improvement with practice and feedforward planning (44, 45, 75–77). Similarly, it was found that people with PD react differently to planned vs. unplanned stopping during gait, whereas healthy controls do not. Patients with PD are better able to stop during planned scenarios, suggesting that feedforward modulation plays an effect (78). Specific defects in shifting between sets are evidenced by reliance on greater cognitive control (79, 80) and reduced automaticity, which causes dual-tasking deficits during movement (81, 82). Difficulty in acquiring and expressing habitual motor patterns has been shown to lead to impairments expressing automatic components of behavior (83, 84), learning implicitly (60–66, 85), performing an unfamiliar task (86), and combining information from different reference frames in order to guide movement (77, 87). However, while feedforward learning seems fairly intact, if slow, these learned sets may not be retained, further indicating an impairment in switching between motor sets (18, 21). In summary, patients with PD have difficulty rapidly and flexibly switching between motor plans in a reactive or unconscious fashion. Such impairment leads to difficulties in novel tasks, tasks in which external factors may change needed forces and trajectories, and dual-tasks, in which two sets must be held simultaneously and rapidly switched between.

### Factors Affecting Motor Learning in PD

A number of factors related to PD might have a major impact on the severity of impact on motor learning: severity and duration of disease, disease subtype (tremor dominant, PIGD dominant, etc.), amount of degeneration in dopaminergic and non-dopaminergic neurotransmitter systems, and cognitive symptoms. Most of these factors have not yet been well-researched. Research suggests that motor learning worsens with disease severity, though these trends are still characterized as weak to moderate. Worsening cognitive and axial symptoms especially appear to predict a decrease in motor learning ability (24, 30, 32). Axial symptoms in particular have been directly linked to worsening motor learning performance, even independently of overall disease severity (88). Specifically, patients with freezing of gait (FOG) seem to exhibit worse learning, retention, and generalization in motor learning tasks (29, 89, 90), though confounds make it difficult to determine the exact influence of a generally longer disease course and worsened disease severity, fall risk, and cognitive profile associated with FOG (91–93). Subtype of PD, as characterized by symptom profile, may play a role in impact on motor learning (31). Laterality of symptoms of PD may also affect acquisition of motor skills in PD, with left-onset PD being correlated with more errors during task acquisition than right-onset PD (94). However, motor learning is affected early in PD and learning deficits appear to be present on both sides, even before traditional symptoms are evident on the less-affected side (95).

Cognitive features of the disease, such as impairments in memory, attention, and executive function (EF) also play a role in motor learning. Memory and other components of EF are notably affected in PD, even early in the disease and with no apparent cognitive impairment (96, 97). These deficits have been attached to deficits in both motor symptoms (97–100) and in learning (101, 102), demonstrating that more widespread frontostriatal system involvement might be related to defects of motor learning in PD. Many of the specific aspects of EF impacted in PD, including planning and attentional deficits, information organization and retrieval, task-switching and establishment of a motor set, feedback-based learning, and sensitivity to interference during learning, directly affect the ability of individuals with PD to learn and modulate motor patterns (97, 98, 103, 104). Dual-tasking may be especially affected by these problems (105). Studies have shown that procedural and spatial memory is especially affected in PD, while verbal and episodic memories remain largely intact (103, 104, 106, 107), suggesting that areas of cognition associated with motor planning and execution may be preferentially affected by the disease. Deficits within attentional networks, and specifically the connections between the putamen and the motor cortex, prevent the sustained switch from controlled to automatic behavior (108). The close interplay between the cortical and subcortical areas regulating motor, cognitive, and motivational aspects of habitual, automatic movement are dysfunctional in PD (109).

In addition to direct effects of PD on motor learning, it has been suggested that external changes, such as medication and environment can impact the ability of patients to learn, retain, and update motor information. One of the most-researched factors affecting learning in PD is levodopa. While levodopa is remarkably beneficial in treating many aspects of PD, including tremor, rigidity, and bradykinesia, it is thought not to improve, or possibly even to worsen, certain aspects of motor learning. Additional deficits in motor learning while in the “on”-state (that is, while the short-term effects of levodopa are active) have been noted in upper (110) but not lower (111–114) extremity learning. However, differences in study design suggest that this difference may be due to the stage and type of learning studies, with specific portions of the acquisition phase of learning most affected (30). Levodopa may attenuate some of the motor deficits in learning while worsening cognitive aspects (115–117). This suggests that while dopaminergic dysfunction plays a role in motor learning, other systems, such as the frontostriatal system, are also key. While not yet well-researched, early data suggests that deep brain stimulation (DBS) might have a positive effect on learning (118). Environmental and task characteristics may also significantly affect ability to learn in PD. One study found that task difficulty significantly affected the ability of people with PD to learn relative to healthy controls. Dual-task and reactive tasks significantly reduced or even eliminated the ability of PD subjects, but not controls, to learn and retain improvements (119).

Increasing the amount of external information provided to individuals with PD, such as through cueing, can significantly improve acquisition, automaticity, and retention of motor tasks, even once cues are removed (67, 120). The beneficial effects of cueing on gait and motor learning (121, 122) in PD are likely largely caused by addressing cognitive factors of the disease, such as attention (71). By directing attention to the motor action being practiced and giving feedback about its correctness, cueing can facilitate feedforward learning. Reward and motivational pathways are also likely affected. PD is known to cause apathy, blunting subjective value of reward (123). This leads to a reduced tendency to evaluate and monitor outcomes and negatively impacts feedback-learning. By providing an external, objective reward, cueing may allow PD patients to incorporate the benefits of positive movement patterns (51).

## Effect of Training on PIGD

Based on studies of motor learning in PD, it is expected that training or therapy specifically targeting gait and balance would lead to significant improvement in these areas. However, these improvements might be less drastic, require more repetition, and be less generalizable to other tasks when compared to healthy controls. Current research appears to support this, as will be discussed in the proceeding sections. Still, there is still need for additional research to discern the types, quantity and intensity of training that is most helpful to people with PD, the generalizability of this training to other tasks, the effectiveness of this training during novel or complex tasks, and the effect of both disease and external factors on learning rate and ability. The following sections will discuss results showing the effects both of traditional therapies, such as PT and exercise (when specifically focused on gait and balance practice) and of repeated perturbation training (an experimental concept that attempts to train individuals how to respond to unexpected perturbation by repeatedly eliciting such events) on gait and posture in PD.

### Physical Therapy and Exercise Therapy

Studies of motor learning indicate that repeated and continued physical training may be the best method to counteract the degradation in motor behavior seen in PD. Physical and exercise therapy have long been noted to be beneficial for patients with PD, improving cardinal symptoms activities of daily living and increasing subjective quality of life (124–134). Recently, technological advancements in therapy delivery (such as through the use of virtual reality and other gamification elements) and ability to calculate objective outcome measures (using IMUs and similar technology) has led to an increasing interest in the effects of such therapies on PIGD features of PD. Many studies have noted improvements in gait and balance following these therapies (11, 127, 135–138). Studies have focused on determining the best types, intensity, and duration of therapy, the effects of the incorporation of technology (both in increasing duration and intensity of exercise and in improving PIGD symptoms), and the duration of sustained effect or need for repeated doses of therapy.

While there is great interest in the types of exercises and techniques that might be best applied to improve specific features of PD, controlled research studies documenting the comparative successes of different options are still relatively few in number. They have mostly utilized small sample sizes and have had somewhat conflicting results. Direct comparisons between studies are made more difficult by the lack of homogeneity between treatments (an example of differing treatment parameters may be seen in Table 1 and outcomes are compared in Table 2). Many studies have differing definitions of treatments even when using the same name, and the duration, intensity, and frequency of training differ between studies. This is made more difficult by the fact that most trials compare results only to control groups (usually similar subjects not given any therapy regiment, but sometimes patients provided with therapy not intended to specifically improve balance) instead of other balance interventions. In the future, it will be important to compare intensity-matched treatment options directly in a controlled manner.

**Table 1:** Comparison of study design from studies regarding the effect of therapy and training on quantitative measures of gait and balance.

**Therapy type**	**Sample size (per group)**	**Treatments**	**Control/Comparison groups**	**# of Weeks**	**# of Sessions/Week**	**Session duration**
Strength/resistance training	10–15 (139, 140)	High-intensity quadriceps (129)	Exercise (129, 141)	4 (140)	1–2 (141–143)	40–60 min (12, 129, 139–142)
	20–25 (12, 129, 142, 143)	Lower limb (12, 139–142)	Multi-component (12, 139, 141, 142)	7 (142)	3 (129, 140)	60–90 min (143)
	65–70 (141)	PRE (143)	Balance (142)	12 (12, 129, 139)	3–5 (12, 139)	
			RPT (140)	24 (141)		
				104 (143)		
Gait training	<10 (126)	Treadmill walking (126, 144–154)	Overground walking (146, 155, 156)	1 (149)	1(149)	20–30 min (120, 126, 145, 149, 154, 155, 157–161)
	10–15 (145–147, 150, 152, 156, 157, 162–166)	Robot-assisted (151, 153, 167)	Exercise/Conventional therapy (147, 149, 164, 165, 167)	3 (120)	2 (154, 155)	40–60 min (144, 147, 148, 151, 153, 156, 163–165, 167)
	15–20 (149)	BWSTT (160, 161, 163–165)	Tango (150)	4 (151, 153, 157, 159–161, 163, 164, 167)	2–3 (152)	Progressive (146, 152, 166)
	20–25 (144, 148, 153, 159–161, 167, 168)	Backward gait (156)	Stretching (150)	5 (146, 152)	3 (120, 144–148, 156–158, 163–166)	Not given (150)
	150–160 (120)	Cued gait (120, 145, 152, 155, 157–162)	Education/Normal Treatment (160, 161, 165)	6 (126, 144, 145, 155, 166)	4 (126, 160, 161)	
		Cued treadmill walking (159)	RPT (154)	8 (154, 156, 158, 165)	5 (151, 153)	
			None (120, 126, 144, 148, 149, 153, 156–158, 166)	12 (148, 150)	6 (167)	
				24 (147)	7 (159)	
					Not given (150)	
Balance training	<10 (169, 170)	Cued (169, 171)	Exercise (172)	4 (171)	2 (142, 173, 174)	20–30 min (170, 171, 173)
	10–15 (171, 173, 174)	Weight-shift (142, 174)	Resistance (142)	6 (169, 173)	3 (13, 169–172, 175)	30–40 min (169)
	20–25 (142, 175)	Sensory perturbation (13, 170, 172, 173, 175)	Balance + resistance (170)	7 (13, 142, 172)		40–60 min (13, 142, 172, 174, 175)
	30–35 (13)	Virtual reality (173)	Home-based balance (13, 174, 175)	8 (174, 175)		
	35–40 (172)		No training (171, 173)	10 (170)		
			None (169)			
Multi-component training	<10 (170)	Balance + resistance (170)	Balance (170)	3 (176)	1–2 (141, 143, 177)	40–60 min (12, 139, 141, 170, 177, 178)
	10–15 (143, 177, 178)	Treadmill + obstacles + balance (178)	Resistance (12, 137, 139, 141)	4 (168, 178)	3 (170, 178)	60–120 min (143, 168)
	25–30 (12, 139)	Gait + balance (12, 139, 168, 176)	Stretching (141)	10 (170, 177)	3–5 (12, 139)	Not given (176)
	30–35 (168, 176)	mFC (177)	No training (168, 177, 178)	12 (12, 139)	3–14 (168)	
	65–70 (141)	Tai chi (141, 177)	None (176)	24 (141)	6 (176)	
				104 (143)		
Home-based gait training	10–15 (179)	Home-based cueing (179, 180)	Conventional gait and cognitive (in-home) (181)	2 (179)	3 (180)	20–30 min (179–181)
	20–25 (180)	Walking (180)	None (179)	6 (180, 181)	4 (181)	
	55–65 (181)	Dual-tasking (181)			7 (179)	
Home-based balance training	10–15 (162, 174, 182–184)	Sensory perturbation (175)	Therapist-guided balance (172, 174, 175)	6 (182–184)	2 (174, 182, 183, 185)	20–30 min (185)
	20–25 (175)	Tailored exercise (162)	Exercise (182, 183, 185)	7 (172)	3 (140, 172, 175, 184)	40–60 min (140, 172, 174, 175, 182–184)
	35–40 (172)	Wii Fit (172, 182–185)	Education (182, 183)	8 (174, 175)	Not given (162)	Not given (162)
		Kinect (174)	None (162, 184)	10 (162)		
				12 (185)		
Repeated perturbation training (RPT)	<10 (15, 186)	Postural perturbation (16, 187)	Treadmill walking (14, 154)	1 (14, 186, 187)	1 (14, 186, 187)	20–30 min (14, 16, 154)
	10–15 (16, 140, 187)	Treadmill perturbation (14, 15, 154)	Resistance (140)	2 (16)	2 (154)	40–60 min (15)
	20–25 (14, 154)	Step training (140, 186)	No training (15)	4 (140)	3 (15)	Not given (186, 187)
			None (16, 186, 187)	8 (15, 154)	14 (16)	

*BWSTT, body-weight supported treadmill training; PRE, progressive resistance exercise; mFC, modified fitness counts*.

*This table documents the study characteristics of each therapeutic study reviewed for this paper. Papers were characterized by type of therapy provided, and approximate sample size of each group per study (many studies had slightly unequal group sizes), control or comparison groups utilized, number of weeks and sessions of therapy and the duration of each therapy session were noted. Both the therapeutic method used and the duration/intensity of therapy varied widely between studies, showing a need for more standardized protocols in the future*.

**Table 2:** Quantitative gait and balance outcomes from training studies.

**Therapy type**	**Results demonstrating improvement**	**Results not showing improvement**	**Ambiguous/Conflicting results**
Strength/resistance training	Walking velocity (12, 129, 139–141, 143)	Stride length (139, 140, 143)	
	Cadence (143)	Double-support time (143)	
	TUG (129, 141, 142)	Dynamic posturography (140, 142)	
	FAB (142)	CGI (142)	
		ABC (139)	
Gait training	Walking velocity (120, 126, 144, 146, 148, 150–152, 154–159, 161–165, 167)	ABC (145)	Gait symmetry (161, 165)
	Stride/step length (126, 144, 146–148, 150–152, 155–159, 161, 163–165)	Fall frequency (145)	Gait variability (126, 144, 148, 162)
	Kinematic analysis (149, 153, 155)	Mini-BESTest (154)	FOGQ (152, 153, 159)
	FOG Assessment (166)		Cadence (144, 146–148, 152, 156, 158)
	Walking distance (timed) (144, 154, 155, 159, 161, 162)		TUG (146, 152, 154)
	Static posturography (146)		
	Dynamic posturography (145, 160)		
	Sit-to-stand (165)		
	Double support time (148, 149, 162, 165)		
	POMA (155, 160, 167)		
	BBS (145, 155, 160)		
	DGI (155)		
	RST (145)		
Balance training	Cadence (175)	Double support time (175)	TUG (142, 169, 174)
	Stride length (175)	FAB (142)	Dynamic posturography (13, 142, 174)
	FGA (175)	CGI (142)	Fall frequency (13, 172)
	Static Posturography (171)		ABC (13, 169, 172, 175)
	Sit-to-stand (171)		
	BBS (13, 169, 172, 174)		
	Walking velocity (172, 175)		
	DGI (172)		
Multi-component training	Kinematic analysis (176)	Double-support time (143)	Dynamic posturography (170, 178)
	Cadence (143)	DGI (178)	Walking velocity (12, 139, 141, 143, 168, 178)
	Fall frequency (12)		Step/stride length (139, 141, 143, 168)
	Dynamic posturography (12, 141)		ABC (139, 178)
	PPT (168)		BBS (177, 178)
	Turning (168)		
	TUG (141, 177)		
	Walking distance (timed) (177)		
Home-based gait training	Walking velocity (179, 180)	Gait variability (181)	FOGQ (179, 180)
	Mini-BESTest (180)	Walking distance (timed) (180)	
	Stride length (179, 181)	Falls Efficacy Scale (180)	
	Cadence (181)		
Home-based balance training	Stride length (175, 183)	Double support time (175)	ABC (172, 175, 184)
	FGA (175, 183)	Stride velocity (175)	
	TUG (182, 184)	Cadence (175)	
	Falls efficacy scale (182)	Fall frequency (172)	
	Walking velocity (172, 183, 184)	Walking distance (timed) (185)	
	Sit-to-stand (184)		
	BBS (172, 185)		
	CBM (184)		
	Static Posturography (184)		
	Dynamic posturography (182, 184)		
	POMA (184)		
	DGI (172)		
	Obstancle clearance (182)		
	Dynamic balance (162, 182)		
Repeated perturbation training (RPT)	Step initiation (16, 186)	Static posturography (14)	Dynamic balance (15, 140, 187)
	Compensatory step length (16)	FOG (15)	
	Walking velocity (14–16, 140, 154)	Mini-BESTest (154)	
	Walking distance (154)		
	Gait variability (14)		
	Stride/step length (15, 16, 140)		
	Cadence (15, 16)		
	Double support time (16)		
	Fall frequency (15)		
	TUG (154)		

*FGA, functional gait analysis; ABC, activities-specific balance confidence scale; TUG, timed-up-and-go; POMA, Tinetti performance oriented mobility assessment; CBM, community balance and mobility assessment; FOGQ, freezing of gait questionnaire; BBS, Berg balance scale; DGI, dynamic gait index; RST, rapid step-up test, FAB, Fullerton advanced balance scale; CGI, clinical global impression scale; PPT, physical performance test*.

*This table shows the aspects of gait and balance found to be helped or not to be helped by different types of therapy. The studies that researched each aspect of gait and balance are recorded*.

Research from motor learning shows that training should ensure enough repetition to allow adequate time for feedforward learning. Introducing exercises using scaffolding in the form of physical assistance, cueing, or enhanced information that is initially given but progressively removed may prove useful. It is also important that exercises be individually relevant but collectively diverse enough to provide meaningful change in multiple aspects of PIGD (188). The use of technology in training of gait and balance for PD has been of interest. Several studies have utilized gamification of training, technology to assist, resist or create movements or perturbation, augmented feedback and VR (119, 121, 139, 144, 155, 169, 172, 173, 179, 180, 182, 183, 189). Cueing in particular shows promise and is an important area of research (120, 145, 155, 157–162, 171, 173, 190).

The main therapy options that have been researched related to improvement of PIGD symptoms of PD are balance training, treadmill training, and resistance or strength training. Generally, strength training, including that focusing on the lower-limbs, has been found to be less likely to improve features of PIGD and is sometimes used as a control group in studies focusing on gait and balance therapies. However, a study comparing the effects of 7 weeks (2 h-long sessions per week) of resistance vs. balance training found that resistance training but not balance training contributed to significant improvements in the Fullerton Advanced Balance (FAB) scale and Unified Parkinson's Disease Rating Scale (UPDRS) scores, though between-group statistical comparison did not differ between the therapies (142). Gait speed and TUG have also shown improvement (129, 191).

A large number of studies regarding the effects of therapy of gait and balance have been related to the use of treadmill therapy (192, 193). Improvements in gait speed, stride length, and symptomatic scales have been noted both immediately and in the long-term following treadmill therapy (126, 146–148, 167). Additionally, some studies have noted improvement in post-urography following treadmill training, suggesting some level of generalizability (146). Improvements appear to be obtained long-term (146). These changes were noted in some studies after only a single session of training. Adverse events were not observed within these studies, and patients were able to achieve a high-intensity of training (149, 192). Treadmill training utilizing virtual reality to simulate dual-tasks and obstacles has been found to improve gait measures under diverse conditions (144). Studies also showed that treadmill therapy compared favorably to other therapy techniques. Pohl et al. comparing speed-dependent treadmill training, limited progressive treadmill training, conventional gait training, and non-intervention controls, found that treadmill training significantly improved gait parameters compared to both controls and conventional gait training (149). However, Myers et al. found that, while treadmill therapy, tango dance, and guided stretching all improved walking velocity and stride length, there was no difference between the three groups (150). Sale et al. found that robot-assisted walking, but not traditional treadmill training, improved gait velocity and stride length, though this result conflicts with all other such studies (151).

The addition of body-weight support to allow for increased intensity of training may help to increase the effect of treadmill therapy (151, 192). Miyai et al. reported that body-weight supported treadmill training significantly improved UPDRS scores, ambulation speed, and number of steps needed to complete a 10-meter walk when compared to traditional physical therapy (163, 164). Another study analyzing the effect of high-intensity body-weight supported treadmill training vs. lower intensity exercise or education, found that, while all three groups improved UPDRS scores, only the high-intensity treadmill training significantly improved gait measures (165). Cueing also appears to improve results of treadmill training (152).

Other types of gait therapy have also shown to be effective. Overground walking, both backward and forward, have been evaluated and found to improve gait characteristics (156). Cued gait training appears to increase stride length, walking speed, dynamic post-urography, and Berg Balance Scale scores (120, 145, 155, 157–159). Wearing a home-based cueing system for as little as 30 min per session has been found to increase gait velocity and stride length and improve FOG (179, 180). Home-based dual-tasking therapy has been shown to improve stride length and cadence (181). Robotic-assisted gait therapy, in which a wearable robot assists in locomotion during training has also been shown effective in improving mobility (153, 160, 167). Studies comparing robot-assisted gait training with other gait therapies found than robot-assisted training may actually be more effective than treadmill therapy (153) and overground gait training with verbal cueing (160, 161).

Balance therapies utilizing static and dynamic stance are common and appear to be effective in improving many gait and balance measures. Atterbury et al. found that both therapist-led and home-based balance training improved walking velocity, cadence and stride length and Berg Balance Scale scores, though therapist-led therapy was more effective (175). A study comparing balance training with augmented feedback to a control group of lower-limb strength training found that balance training more significantly improved measures of limits-of-stability, one-leg stance, and gait, and was the only group to improve balance confidence (139). Cued sit-to-stand training has also been shown to improve balance and stability (171). Cued training during practice on courses involving turns reduced FOG (166). A study comparing the training of compensatory stepping using a dancing game with visual cues to a control group receiving strength training found that the step training significantly improved reaction time, movement velocity, limits of stability and UPDRS gait sub-score relative to the control group, but both groups improved gait velocity and only strength training improved cadence during gait (140).

Home-based balance therapies have often been researched in recent years, often using virtual reality and video game technology, such as Wii Fit (194). This type of training has been found to be effective in improving multiple features of gait and static stance, suggesting that home-based therapies may be an effective supplement or alternative to in-person therapies (172, 182–185). Dynamic balance is also improved, as measured by the Sensory Organization Test (SOT) (162). A trial using the Xbox Kinect found comparable improvements in TUG and Berg balance scores and a significantly greater improvement in dynamic stability compared with traditional balance training (174). Yen et al. randomized patients to receive either virtual-reality based balance training, traditional balance training, or no therapy (control group) found that both training groups displayed improvements dynamic post-urography (SOT) (173). A balance training program utilizing audio-biofeedback was also found to improve Berg balance scores and TUG (169).

Combination therapies have also been utilized, but experimental confounds have made it somewhat difficult to interpret these results. Hirsch et al. compared combination balance and resistance therapy vs. balance training. They found that, while computerized dynamic post-urography measured by the SOT and muscle strength improved in both experimental groups, combination therapy demonstrated larger improvements. However, patients in the combination group were given 15 min of resistance therapy three times a week in addition to the thrice-weekly 30 min balance training sessions experienced by both groups, so it is difficult to determine how much of this greater effect is due to combination therapy vs. greater duration of therapy in the combination group. In addition, no comparison was made to a group receiving resistance therapy alone (170). A study which assigned the experimental group to complete balance and gait training and the control group to complete an equal number of sessions of strength training found that the experimental group was less likely to fall, fell less often and demonstrated greater reduction in postural response length and increase in stride length compared to controls (12). Gait and balance therapy has also been found to improve gait kinematics (176), gait velocity, step length, turning, and Physical Performance Test scores (168). A study comparing resistance with a multi-modal training program found that both groups similarly increased velocity and cadence of off-medication gait and plantarflexion strength, though other measures of spatial gait parameters were unaffected (143). Smania et al. found that a balance training program with locomotor training, but not a control group exposed to full body active joint mobilization, muscle stretching, and muscle coordination exercises, showed improvements in several measures of balance and balance confidence (13). Tai chi, a martial art form that addresses many components of gait and balance, has also been used with some success (141, 177). However, not all multi-component therapies have been found to be successful. Hirsch et al. found that a 4 weeks therapy utilizing treadmill training, obstacle course training, and balance training did not significantly improve any features of gait relative to a treatment-free control group (178). This could be due to short therapy duration, low intensity of therapy, lack of sensitive outcome measures, or small sample size.

### Repeated Perturbation Training

Traditional physical and exercise therapies have successfully improved the gait and balance of patients with PD. Motor learning challenges in PD demonstrate that specific, feedforward practice of events related to PIGD and falls may be even more effective than generic practice or strength training. This evidence has elicited interest in the utilization of repeated perturbation training (RPT) for the improvement of PIGD in PD. Specifically, repeated training of reactions to the types of unexpected perturbations that may lead to falls is thought to show promise in improving balance and decreasing falls in daily life. Because such perturbations take place in a controlled system, with the provision of supports as needed, they can be practiced safely. As noted in the section above, several successful therapy programs have incorporated forms of perturbation training. However, repeated perturbation testing, especially that using technology-assisted protocols, has not yet been widely adopted and research is still needed to determine the types of perturbation that are most helpful. The duration, frequency, and intensity in which these therapy sessions must occur and the generalizability of reactions from practiced perturbation to another novel type also need to be further researched.

Most of the studies of RPT within the PD population have focused on the effects of training during static or dynamic stance. Several studies utilizing the sudden and unpredictable movement of a platform under subject's feet have noted that repeated perturbations of this type generate a training effect, improving subject response in later trials. Visser et al. found that, while patients with PD always differed from age-matched healthy controls in kinematic and surface EMG measures of their response to the sudden tilting of the platform they are standing on, individuals with PD do show significant improvement in these balance reactions following training (187). van Ooteghem et al. achieved similar results when patients attempted to maintain balance while the support platform oscillated at random amplitudes (which repeated in sequence unknown to the subjects). People with PD improved reactions to this perturbation, shifted from feedback to feedforward mechanisms as a strategy for improving performance, and generalized learning to a different pattern of oscillatory movements. More widespread generalizability to other types of perturbation (e.g., sudden translation or other movement) was not tested (195).

Several studies have tested improvement of compensatory steps as a reaction to external perturbation. A study utilizing mechanically applied perturbation using a weighted pulley system attached to the shoulders found that the length of compensatory steps increased and time to step initiation shortened with repetition. Interestingly cadence and step length during gait also improved, implying some level of generalizability to normal gait (16). Peterson et al. found that people with PD are capable of motor learning when repetitive forward and backward translations are applied to the support surface and that these improvements were retained a day later, but they did not generalize to lateral translations. Interestingly, the patients in this study were found to exhibit the most improvement in the first trial, while most studies found that individuals with PD need several trials before motor learning occurs (88). A study that utilized a combination of treadmill therapy and RPT in which perturbations found that this training resulted in a reduction in falls and improvements in gait and dynamic balance (15).

Only a few studies have previously reported on the ability of perturbations during dynamic gait to improve PIGD in PD. Roemmich et al. found that people with PD are able to adapt when split belts of a treadmill were rotated at differing speeds beneath them, and aftereffects and shorter re-adaptation indicated that these changes might be retained (17). Klamroth et al. showed that perturbation treadmill training utilizing tilt, in comparison to traditional treadmill walking, was able to induce adaptation and improve gait velocity and variability. A small effect was noted in static postural control, which could indicate possibility for generalized effect (14). Steib et al. found that treadmill perturbation therapy can induce changes in dynamic balance and gait, generating greater effects than a treadmill training control group (154). While these studies have been promising, much more research is needed to determine the clinical efficacy of RPT for gait, including the number, duration, and intensity of sessions needed, the duration in which effects of treatment may last, the types of changes induced by various types of perturbations, and the effect these changes have on decreasing both real-life fall incidents and simulated falls under different conditions. Perturbation during step initiation could also be an area of interest, possibly for reducing start hesitation, a form of FOG. Rogers et al. found that perturbation, either through a drop or an elevation of the support surface under the initiating foot, timed directly at the point of step initiation, elicited adaptation of stepping response to preserve stability and that this augmentation could improve postural and locomotor coupling (186).

## Conclusions and Future Directions

Research related to motor learning in PD and effects of therapy programs in PD populations indicate that patients with PD are capable of motor learning. However, this learning may be slower, of smaller magnitude, and less generalizable relative to healthy individuals. A combination of sensory degradation and neurological degeneration (affecting physical, attentional, and learning capabilities) may make adaptation to feedback difficult, forcing patients with PD to rely on feedforward methods of learning. Gait and balance are inherently subject to constantly changing environmental and other conditions, making feedforward control difficult; however, training may improve this ability. In order to more effectively train individuals with PD in forming feedforward strategies, it is important that therapies are targeted at the symptoms they intend to improve, that patients be given ample feedback about their current movement patterns and needed improvement, and that duration, intensity, and timing of therapy sessions be set to maximize motor learning. In this case, frequent and intense workouts, with duration long enough to achieve learning effect without resulting in fatigue, would be ideal. Therapies should also attempt to incorporate additional feedback (possibly in the form of verbal prompts, cues, or augmented reality systems).

Current research has shown that therapy programs specifically targeted to improve factors related to gait and balance can significantly improve these features. Resistance training, balance training, treadmill training, and repeated perturbation training have all been shown to exhibit these effects. However, more research is still needed to determine what therapies are more effective for specific aspects of PIGD (e.g., resistance training may help to increase voluntary muscle activation and movement amplitude and improve bradykinesia while RPT may reduce falls or FOG) and what aspects or exercises of these therapy modalities are most helpful for these improvements. Efforts should be made to standardize treatments and outcome measures from all treatment groups so that direct comparisons can be made. Studies in which duration and intensity of different types of therapies are as well-matched as possible are urgently needed. The study of combination therapies, the use of varying types of exercises in order to increase different aspects of gait and balance, and the use of enhanced feedback, assistance and body support, and cueing systems will be instrumental features of study. The generalizability of therapeutic effect is also a concern; for example, eliciting perturbations during stance may not help to improve reactions to perturbations during gait. It is important to research the generalizability of each type of training in order to better target training to treat those specific aspects of PIGD most bothersome to the patient. This would maximize the benefit derived from therapy while minimizing duration, avoid patient fatigue and more reasonably adhering to insurance coverage as the research moves into the clinical sphere.

Patients with PD seem to rely largely on feedforward learning, even in cases, such as response to external perturbation or changing conditions. The effects of situations that might interfere with learning feedforward movement patterns, such as dual-tasking or operating under reduced sensory feedback, need to be studied. It has been seen that individuals with PD have special difficulty with certain tasks, including gait and balance under these conditions. Further research could reveal interesting information about motor learning and maintaining and updating motor loops in PD. The use of augmented feedback to induce learning of proper patterns could help to facilitate the transfer of feedforward methods learned to situations that are likely to induce falls and is also of interest for research. Because gait and balance both involve constantly changing conditions and some degree of dual tasking is quite common, assisting the improvement of these skills could be vital to reducing falls. As of yet, little research has focused upon this training or upon the limits of feedforward control in managing PIGD.

Research into motor learning and control can directly facilitate improvements in therapy. While preliminary, current research suggests that as much exercise as possible, both through therapy and through continued home and gym exercise, should be recommended. Therapy should address as closely as possible the active concerns patients with PD are experiencing (such as balance, treadmill, or lower-limb focused resistance training for patients known to be experiencing severe PIGD). At present, the exact exercises prescribed are largely variable, but the addition of more evidence-based research in the field will help to determine the best potential options based on patient profile. A high level of feedback is recommended, possibly through the use of VR, cueing or other augmented feedback technologies.

## Author Contributions

MO was responsible for compiling the sources used within this review and writing the drafted manuscript. TL and AL provided additional articles, discussed possible implications, and revised the manuscript.

### Conflict of Interest Statement

The authors declare that the research was conducted in the absence of any commercial or financial relationships that could be construed as a potential conflict of interest.

## Appendix

### Article Selection Methods

Keyword searches within the PubMed and Google Scholar databases were used to identify articles of interest. Only articles written in or translated into English with full text available were considered. Additional papers of relevance were selected from the citations given by those papers initially found during the literature search.

For section Motor Learning in PD, articles related to “Motor” + “Postural learning” or “Implicit learning” or “Explicit learning” or “learning” + ”Parkinson's disease” or “Parkinson disease,” “Memory” or “Attention” or “Executive function” + “Motor learning” + ”Parkinson's disease” or “Parkinson disease,” “Feedback” or “Perception” + “Motor learning” + ”Parkinson's disease” or “Parkinson disease,” and “Motivation” + “Motor learning” + ”Parkinson's disease” or “Parkinson disease” were searched for. Abstracts were read to ascertain whether motor learning deficits of idiopathic PD in human subjects were discussed. For the relevant titles, the full papers were then read to determine methods used and types and definitions of learning analyzed. Because of the wide focus of titles related to motor learning, which considered varying definitions and perspectives of learning and the effects of various other cognitive and perceptual systems, these articles were not synthesized into a systematic review but grouped and discussed with other similar research.

Within section Effect of Training on PIGD, searches were made for “Physical therapy” or “exercise therapy” + “gait” or “balance” + “Parkinson's disease” or “Parkinson disease,” “therapy” or “training” + “gait” or “balance” + ”Parkinson's disease” or “Parkinson disease,” “Perturbation” or “Perturbation training” or “Perturbation training” + “gait” or “balance” + ”Parkinson's disease,” and “Virtual reality training” + “gait” or “balance” + “Parkinson's disease” or “Parkinson disease.” Articles that applied a specific non-pharmaceutical treatment or treatments involving training or therapy, with quantitative measures of overground gait and/or standing balance changes as a primary outcome measure, were selected through abstract review. Perturbation-based studies were also considered if they measured learning effect to trained or generalized perturbations. Review focused on articles relating to individual therapies, not class-based techniques. Articles were not utilized if pharmaceutical, surgical, or other treatments were tested in conjunction with therapy. Only controlled studies, not case studies or series, were considered. Full articles of selected abstracts were then read to ensure relevance. While training methods, intensity, and durations varied too widely for statistical comparison, studies were analyzed and compared (as shown in Tables 1, 2) to note basic trends in effective treatments. Reviews of therapy and training methods in relation to gait and balance were also considered and conclusions discussed in the body of the paper, though only research studies were included in the Tables.
